# Viscoelastic hydrogel combined with dynamic compression promotes osteogenic differentiation of bone marrow mesenchymal stem cells and bone repair in rats

**DOI:** 10.1093/rb/rbae136

**Published:** 2024-11-23

**Authors:** Chao Yang, Wenbin Cai, Pan Xiang, Yu Liu, Hao Xu, Wen Zhang, Fengxuan Han, Zongping Luo, Ting Liang

**Affiliations:** Department of Orthopaedic Surgery, The First Affiliated Hospital of Soochow University, Suzhou, Jiangsu 215000, PR China; Department of Orthopaedic Surgery, The First Affiliated Hospital of Soochow University, Suzhou, Jiangsu 215000, PR China; Department of Orthopaedic Surgery, The First Affiliated Hospital of Soochow University, Suzhou, Jiangsu 215000, PR China; Medical 3D Printing Center, Orthopedic Institute, Department of Orthopedic Surgery, The First Affiliated Hospital, Suzhou Medical College, Soochow University, Suzhou, Jiangsu 215000, PR China; Medical 3D Printing Center, Orthopedic Institute, Department of Orthopedic Surgery, The First Affiliated Hospital, Suzhou Medical College, Soochow University, Suzhou, Jiangsu 215000, PR China; Medical 3D Printing Center, Orthopedic Institute, Department of Orthopedic Surgery, The First Affiliated Hospital, Suzhou Medical College, Soochow University, Suzhou, Jiangsu 215000, PR China; Medical 3D Printing Center, Orthopedic Institute, Department of Orthopedic Surgery, The First Affiliated Hospital, Suzhou Medical College, Soochow University, Suzhou, Jiangsu 215000, PR China; Department of Orthopaedic Surgery, The First Affiliated Hospital of Soochow University, Suzhou, Jiangsu 215000, PR China; Medical 3D Printing Center, Orthopedic Institute, Department of Orthopedic Surgery, The First Affiliated Hospital, Suzhou Medical College, Soochow University, Suzhou, Jiangsu 215000, PR China

**Keywords:** viscoelasticity, dynamic mechanical loading, bone regeneration, BMSCs, TRPV4

## Abstract

A biomechanical environment constructed exploiting the mechanical property of the extracellular matrix and external loading is essential for cell behaviour. Building suitable mechanical stimuli using feasible scaffold material and moderate mechanical loading is critical in bone tissue engineering for bone repair. However, the detailed mechanism of the mechanical regulation remains ambiguous. In addition, TRPV4 is involved in bone development. Therefore, this study aims to construct a viscoelastic hydrogel combined with dynamic compressive loading and investigate the effect of the dynamic mechanical environment on the osteogenic differentiation of stem cells and bone repair *in vivo*. The role of TRPV4 in the mechanobiology process was also assessed. A sodium alginate–gelatine hydrogel with adjustable viscoelasticity and good cell adhesion ability was obtained. The osteogenic differentiation of BMSCs was obtained using the fast stress relaxation hydrogel and a smaller compression strain of 1.5%. TRPV4 was activated in the hydrogel with fast stress relaxation time, followed by the increase in intracellular Ca^2+^ level and the activation of the Wnt/β-catenin pathway. The inhibition of TRPV4 induced a decrease in the intracellular Ca^2+^ level, down-regulation of β-catenin and reduced osteogenesis differentiation of BMSCs, suggesting that TRPV4 might be the key mechanism in the regulation of BMSC osteogenic differentiation in the viscoelastic dynamic mechanical environment. The fast stress relaxation hydrogel also showed a good osteogenic promotion effect in the rat femoral defect model. The dynamic viscoelastic mechanical environment significantly induced the osteogenic differentiation of BMSCs and bone regeneration, which TRPV4 being involved in this mechanobiological process. Our study not only provided important guidance for the mechanical design of new biomaterials, but also provided a new perspective for the understanding of the interaction between cells and materials, the role of mechanical loading in tissue regeneration and the use of mechanical regulation in tissue engineering.

## Graphical Abstract

**Figure:**
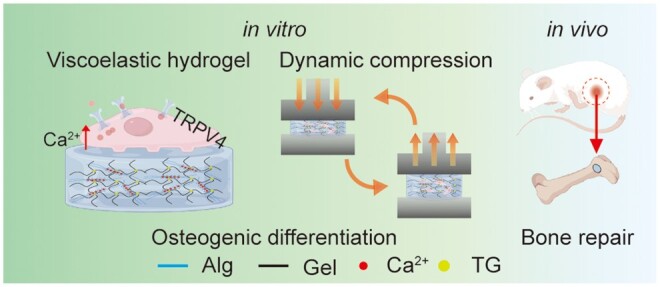

## Introduction

Bone injuries are common events whether they result from medical conditions or accidents. However, the reconstruction of a significant bone loss represents a global challenge [1, 2]. Although self-donated bone grafts are the preferred method of bone restoration, their use is limited by the inadequate number of donation sites. On the other hand, allografts and xenografts can serve as alternatives, although they carry risks of disease transmission and immune rejection [3, 4]. This implies limitations to the existing methods for treating bone injuries and the need to develop more effective treatments to address this challenge. Biomaterials have been used in bone regeneration with positive results [5–7]. Several key factors regulate cell fate, including growth factors, extracellular matrix ligands and mechanical factors. The application of these findings in tissue engineering has contributed to the advancement of bone injury treatment [8–10].

The extracellular matrix is a flexible scaffold consisting of proteins and biopolymers, providing physical support and biochemical signals to cells in tissues [11]. The use of biomaterials to mimic the characteristics of the extracellular matrix to provide cells with a near-natural living environment should be more effective in promoting bone defect repair. In recent years, the influence of the mechanical properties of the extracellular matrix on cell migration, spreading, differentiation, stemness of stem cells and proliferation has been extensively investigated [12–17]. The biological extracellular matrix is not purely elastic but rather viscoelastic with two mechanical properties: energy dissipation and time-dependent mechanical response (stress relaxation and creep). Thus, researchers have paid more and more attention to the influence of viscoelasticity on cell behaviour by constructing various biomaterials, such as polyacrylamide and sodium alginate, to independently regulate the viscoelasticity [18–20]. Cells on viscoelastic substrates with low elastic modulus showed larger cell spreading area, stronger nuclear localization of yes-associated protein (YAP) and stronger osteogenic differentiation than those on purely elastic substrates with high elastic modulus [19, 21]. However, the viscoelasticity on a high elastic modulus matrix does not have these effects [18, 22]. Nevertheless, mesenchymal stem cells encapsulated in a high-viscosity substrate not only show enhanced stemness but also an increased ability to differentiate into cartilage and bone tissues, as demonstrated by *in vivo* experiments [20, 23–25]. Studies on the biological effects of viscoelasticity suggest that the inhibition of Rho GTPase and the increased nuclear localization of YAP may be related to the biological effects of viscoelasticity in a 2D culture environment [19, 21]. In contrast, the aggregation of adhesion ligands, the activation of TRPV4 and the ROCK signalling pathway are involved in a 3D culture setting [20, 23, 25]. Despite the progress made in this field, many unclear aspects remain on the impact of viscoelasticity on cells. A deeper understanding of these biological effects might revolutionize tissue engineering, especially in the treatment of bone defects.

The dynamic mechanical loading is another key factor involved in the osteogenic differentiation of stem cells. Proper functional exercise and sports are a natural way to strengthen bones without side effects. Mechanical stimulation has long been considered a safe and reliable approach to strengthen bones and prevent osteoporosis, as well as increase the repair of bone defects. *In vitro* cell experiments, and animal experiments clearly showed that mechanical force directly stimulates bone cells through different signalling pathways, especially promoting the proliferation, differentiation and gain of osteoblasts in the bones [26, 27].

From a biomechanical point of view, osteoblasts, osteoclasts and osteocytes are constrained by bone deformation due to their attachment to the bone. The deformation generated by the bone stimulated by mechanical forces, defined as deformation/initial length, is always within a normal physiological range, and its final deformation is 1.5% at the time of fracture [28]. However, the deformation range used in most *in vitro* cellular and molecular experiments is much greater than 1.5%, usually between 5% and 20% [29–32]. Therefore, the study of the osteogenic effect of 1.5% small deformation on cells might provide a basis for the mechanical regulation of exercise rehabilitation and bone tissue engineering *in vivo*, with important clinical significance. A significant osteogenic effect with small deformation can be obtained if the extracellular matrix/scaffold material is viscoelastic combined with dynamic mechanical loading, which might provide an important research basis for the mechanical design of bone tissue engineering.

TRPV4 is a calcium (Ca^2+^) ion channel [33], which was originally thought to be involved in the signal transduction of osmotic stress. However, recent studies showed that TRPV4 induce an important effect in bone development. Indeed, the osteogenic differentiation of BMSCs in TRPV4-deficient mice is decreased. TRPV4 enhances the osteogenic differentiation in 3D cultures due to viscoelastic-induced cell volume expansion [23, 34, 35]. Notably, TRPV4 is colocalized with structures such as focal adhesion and primary cilia, which are essential for cellular sensing of mechanical signals [36–38]. In addition, TRPV4 mediates the osteogenic differentiation of MSCs induced by fluid shear stress [39]. These findings suggest that TRPV4 acts as a mechanoreceptor that senses the mechanical microenvironment, consequently controlling the osteogenic differentiation of cells. However, the role of TRPV4 in the regulation of the osteogenic MSC differentiation in a dynamic mechanical environment remains unclear.

In this study, sodium alginate–gelatine hydrogels with different stress relaxation times were prepared. The effect of hydrogel viscoelasticity and dynamic compression on the osteogenic differentiation of BMSCs, as well as the mechanism regulating it were investigated *in vitro*. Furthermore, the rat femoral condyle model was used to study the dynamic viscoelastic effect on bone repair *in vivo*. This study aims to address the challenges of bone defect repair. By adjusting the viscoelasticity of hydrogels and dynamic loading conditions, this research explores their effects on BMSC osteogenic differentiation and the underlying mechanisms, providing new insights for improving bone tissue engineering.

## Materials and methods

### Hydrogel preparation

Hydrogel preparation was modified based on the previously reported methods [40]. In brief, the hydrogel consisted of two main components: sodium alginate (low molecular weight: EFL-Alg-50K (EFL, China); high molecular weight: Sodium Alginate 80-120 (FUJIFILM Wako Pure Chemical Corporation, Japan)), and gelatin from porcine skin (Sigma-Aldrich, USA). Sodium alginate and gelatine were mixed at the concentrations specified in Supplementary Table S1 and dissolved in phosphate-buffered saline (PBS) to prepare a sodium alginate–gelatine solution. Next, calcium sulphate dihydrate (Sangon Biotech, China) was dissolved in deionized water at the specified concentrations to obtain a calcium sulphate solution of the desired concentration. TG enzyme solution was prepared by dissolving transglutaminase (Dolai Biotech, China) in deionized water at the specified concentration. A certain volume of the sodium alginate–gelatine solution was added into one syringe, and a specific volume of the calcium sulphate solution and TG enzyme solution was added into another syringe. The two syringes were connected with a female coupler and the syringes were pushed alternately to mix the solutions thoroughly. Finally, the mixed solution was extruded and allowed to sit for a period of time until the mixture solidified into a hydrogel. Concentrations of various components in the hydrogel are detailed in Supplementary Table S1. The appearance of the hydrogel was obtained from the camera.

### Microstructure observation

After preparation, the hydrogels were balanced in MEMα culture medium for 24 h and freeze-dried for 72 h. The hydrogels were then cut open with a blade, the cross-section was adhered to the scanning electron microscope sample stage with conductive glue, followed by gold sputtering treatment with an ion sputter, setting the gold sputtering parameters to a current intensity of 20 mA, continuing to sputter gold for 45 s. Afterward, the microscopic morphology of the hydrogel cross sections was observed with a scanning electron microscope (Quanta 250, FEI, Hillsboro, OR, USA).

### Mechanical characterization

Hydrogel samples with a height of 2 mm and a diameter of 10 mm were used in unconfined compression tests. The samples were compressed at a speed of 1 mm/min until broken by using an electric mechanical testing machine (Shanghai Hengyi Precision Instrument Co. Ltd, China). Young's modulus was obtained by calculating the slope of the compressive stress–strain curve within the strain range of 5–10%. In the stress relaxation measurement, the samples were compressed at a speed of 1 mm/min until the strain of 15%. The strain was then kept constant to obtain the stress relaxation. The stress relaxation time was defined as the time reaching half the value of the maximum stress. Cyclic compression tests were performed in 100 cycles using a speed of 1 mm/min, maximum strain range of 20% and minimum stress of 0 in each cycle.

### Rheological test

Hydrogel samples with a height of 2 mm and a diameter of 20 mm were used in this rheological test by using a rheometer (TA, USA) with a 25 mm diameter plate fixture. Frequency scan parameters were set as follows: temperature of 37°C, a fixed amplitude of 1% and an oscillation angular frequency from 0.1 to 10 Hz.

### Degradation test

The degradation properties of the hydrogel were assessed in Minimum Essential Medium Eagle—Alpha Modification (MEMα medium, Gibco, China) at 37°C. The hydrogel was prepared following the previously described method. The hydrogel was equilibrated in the medium for 1 day to allow full swelling and complete dissolution of the calcium sulphate particles within the hydrogel. Then, the hydrogel was removed from the medium, rinsed with deionized water, freeze-dried and weighed to determine the initial weight (*W*_0_). Subsequently, at different time points, the hydrogel was taken out of the medium, rinsed with deionized water, freeze-dried and weighed to obtain the weight of the hydrogel after different degradation times (*W_t_*). Finally, the weigh percentage was calculated using the following formula: weigh percentage = *W_t_*/*W*_0_ × 100%.

### Cell culture

BMSCs were extracted from the femurs of 4-week-old male SD rats and cultured at 37°C with 5% CO_2_ in MEMα medium, supplemented with 10% foetal bovine serum (Celligent, New Zealand) and 1% penicillin–streptomycin (Procell Life Science&Technology Co., Ltd, China). The medium was changed every two days. The third passage of BMSCs was used for all cellular experiments.

### Live/dead staining

Before cell culture, two kinds of hydrogels were prepared in a 6-well plate and equilibrated in the culture medium overnight. To evaluate the biocompatibility of the hydrogels, cells were seeded onto the hydrogels in the 6-well plate and cultured for 1 day. Staining was performed using the Calcein/PI Cell Viability and Cytotoxicity Assay Kit (Beyotime, China), following the manufacturer's instructions. After staining, all images were captured using the Zeiss Axio Observer.Z1/7 fluorescence microscope (Carl Zeiss, Germany).

### Cytoskeleton staining

Two hydrogels were prepared in a 6-well plate and equilibrated in the culture medium overnight. To assess cell spreading morphology, cells were seeded onto the hydrogels in the 6-well plate and cultured for 1 day. Subsequently, cells were fixed at room temperature with 4% paraformaldehyde for 40 min. After removing the fixative, the samples were rinsed three times with PBS. The samples were then incubated in PBS containing 0.3% Triton X-100 and 3% bovine serum albumin for 1 h to permeabilize the cell membrane and block non-specific protein interactions. The actin filaments were then stained with Actin-Tracker Green-488 (Beyotime, China) for 40 min, and the nuclei were stained with DAPI for 10 min. After staining, all images were captured using the Zeiss Axio Observer.Z1/7 fluorescence microscope (Carl Zeiss, Germany).

### CCK-8 assay

To assess the biocompatibility of the hydrogels, 1 g of hydrogel was soaked in 5 ml of culture medium for one day to obtain the extract liquor from the two types of hydrogels. Cells were cultured in a regular medium and with the two leachates. The effects of the leachates on cell viability were determined on the first day and the third day using the cell counting kit-8 (CCK-8) assay. The 10% CCK-8 (NCM Biotech, China) solution was added to each sample, incubated in the incubator for 1 h, and then the absorbance was measured at a wavelength of 450 nm using a microplate reader (Bio Tek Instruments, USA).

### Cell scratch assay

Before cell culture, two hydrogels were prepared in a 6-well plate and equilibrated in the culture medium overnight. The stainless-steel square columns with wide of 1 mm were sterilized by autoclaving and then placed on the hydrogel surfaces. The cells were inoculated and waited until the hydrogel was covered. The stainless-steel square column was removed and the culture medium was replaced with 1% serum-containing medium. The images were captured at the appropriate times. The scratched area was quantified and the relative migration rate was calculated using Image J by dividing the covered scratch area by the original scratch area.

### Osteogenic differentiation of BMSCs

Two hydrogels were prepared in a 6-well plate and equilibrated in the culture medium overnight. After seeding the cells, the osteogenic differentiation medium was added to induce osteogenic differentiation once the cells reached 80–90% confluence. The osteogenic induction medium was the culture medium containing 10 mM glycerol phosphate disodium salt hydrate (Sigma, USA), 50 μg/ml vitamin C (Sigma, USA) and 100 nM dexamethasone (Sigma, USA).

### ALP staining

After osteogenic induction for 7 days, the cells cultured on two hydrogels were fixed with 4% paraformaldehyde for 30 min and then washed with PBS three times, each time for 5 min. Subsequently, following the manufacturer's instructions, the BCIP/NBT alkaline phosphatase staining kit (Beyotime, China) was used for staining. After staining, photographs were taken using both a smartphone and an inverted microscope (Olympus, Japan). The images captured under the microscope were quantified using Image J.

### Dynamic compressive loading

The BMSCs were digested, centrifuged and resuspended in PBS to prepare hydrogels. The hydrogels for compression were made in a mould to ensure a consistent height of 5 mm. The hydrogels were cultured in a 6-well plate, and after one day, the normal medium was replaced with an osteogenic induction medium. The 6-well plate was placed in a compression apparatus and compressed with a strain amplitude of 1.5 or 5%, frequency of 1 Hz, 2 h a day. mRNA was extracted after three days of loading. The compression device is described in Supplementary Figure S1.

### Application of GSK2193874

To inhibit TRPV4 activity, the osteogenic induction was performed using an osteogenic induction medium containing 100 nM TRPV4 pharmacological inhibitor GSK2193874 instead of a normal osteogenic induction medium.

### qPCR analysis

Total RNA was extracted from cells using the Trizol reagent (Invitrogen, USA) after three days of osteogenic induction following the manufacturer's instructions. Additionally, for cells cultured inside the hydrogel, after 3 days of osteogenic induction, the medium was removed and the hydrogel was washed three times with PBS. Then, the hydrogel was transferred into an EP tube, and 1 ml of Trizol reagent was added, and the hydrogel was crushed using a handheld tissue grinder. Subsequently, the mixture was centrifuged at 12 000*g* for 10 min at 4°C, and the supernatant was transferred into a new 1.5 ml EP tube, and total RNA was extracted according to the manufacturer's instructions. RNA concentration was determined using NanoDrop Microvolume Spectrophotometers (Thermo Fisher Scientific Inc., USA). Subsequently, 1 μg of RNA was reverse transcribed into cDNA using the HiScript IV RT SuperMix for qPCR (Vazyme, China). The cDNA samples were subjected to qPCR using SYBR Green (Bio-Rad, USA) and the CFX96 real-time system (Bio-Rad, USA). The relative expression levels of each gene were calculated using the 2^−ΔΔ^^*Ct*^ method. Primer sequences are shown in Supplementary Table S2.

### Immunofluorescence staining

For the immunofluorescence staining of osteogenic-related proteins, the culture medium was removed after 3 days of osteogenic induction, and cells were fixed with 4% paraformaldehyde for 30 min. Antibodies against RUNX2 (1:200, Affinity, China) and OCN (1:200, Affinity, China) were incubated overnight at 4°C, followed by fluorescence labelling using the appropriate Alexa Fluor 594 secondary antibody (1:500, Yeasen, China). Cell nuclei were stained with DAPI (Beyotime, China). The actin filaments were then stained with Actin-Tracker Green-488 (Beyotime, China), and all images were captured using the Axio Observer.Z1/7 fluorescence microscope (Carl Zeiss, Germany).

For the immunofluorescence staining of TRPV4, after culturing cells on the hydrogel surface for one day, the culture medium was removed, and the cells were fixed with 4% paraformaldehyde for 30 min. Cells were incubated overnight at 4°C with the anti-TRPV4 antibody (1:200, Affinity,), followed by fluorescence labelling using the appropriate Alexa Fluor 594 secondary antibody (1:500, Yeasen, China). The cell nuclei were stained with DAPI (Beyotime, China), and all images were captured using the Axio Observer. Z1/7 fluorescence microscope (Carl Zeiss, Germany).

### Intracellular calcium ion imaging

After the cells were cultured on the hydrogels for one day, the culture medium was removed and the cells were washed three times with PBS. Fluo-4 AM (calcium ion fluorescent probe, 2 mM) (Beyotime, China) was used for staining according to the manufacturer's instructions. All images were captured using the Zeiss Axio Observer.Z1/7 fluorescence microscope (Carl Zeiss, Germany).

### Calcium assay

Hydrogels were prepared in 6-well plates as described previously. Five milliliters of osteogenic induction medium were added to each well. The medium was removed after 1 day. The Calcium Colorimetric Assay Kit (Beyotime, China) was used to measure the calcium ion concentration in each group of culture media according to the instructions.

### Western blot

Total proteins from the cells induced for osteogenesis on the hydrogels for 3 days were extracted using RIPA lysis buffer (Beijing Solarbio Science & Technology Co., Ltd China) according to the manufacturer's instructions. PVDF membranes were performed by incubating with primary antibodies against COL1 (1:1000, Affinity, China), RUNX2 (1:1000, Affinity, China) and GAPDH (1:10000, Abcam, Britain) overnight at 4°C. PVDF membranes were incubated with HRP-conjugated secondary antibodies for 1 h at room temperature, and images were acquired using NcmECL Ultra (NCM Biotech, China).

### Application of hydrogels in bone defect repair

All experimental procedures were approved by the Institutional Animal Care and Use Committee of Soochow University (SUDA20231116A05). A total of 24 male SD rats, 8 weeks old, were used in this study. A bone defect with a diameter of 3 mm and a depth of 3 mm was created on the lateral condyle of the distal femur for each rat. The rats were randomly divided into three groups, with eight rats in each group: Defect group (the defect was rinsed with saline and the wound was sutured); F group (fast stress relaxation hydrogel was placed in the defect); S group (slow stress relaxation hydrogel was placed in the defect). At either the 4th or the 8th-week post-surgery, the rats were euthanized and the femurs were collected for further testing.

### Micro-CT and histological evaluation

The femur samples were fixed with 4% paraformaldehyde and scanned with a micro-CT scanner (Sky Scan 1176, Sky Scan, Aartselaar, Belgium). Bone regeneration was estimated based on bone volume/tissue volume (BV/TV) in the region of the defect. For histological analysis, decalcified samples were embedded in paraffin and sectioned into 6 μm thick slices using a microtome (LEICA, SM2000R). The specimens were then stained with hematoxylin–eosin staining (H&E) and Masson following standard procedures for histological observation.

### Statistical analysis

All data were presented as mean ± standard deviation. All statistical comparisons were performed in Prism 10 software (GraphPad Software, USA). *T*-tests were used to compare two data groups, one-way ANOVA or two-way ANOVA was used to determine statistical differences between multiple sets of data. All cell experiments were conducted with three biological replicates. Differences with a *P* values <0.05 were considered statistically significant.

## Results

### Characterization of sodium alginate–gelatine hydrogel

**Figure 1.**
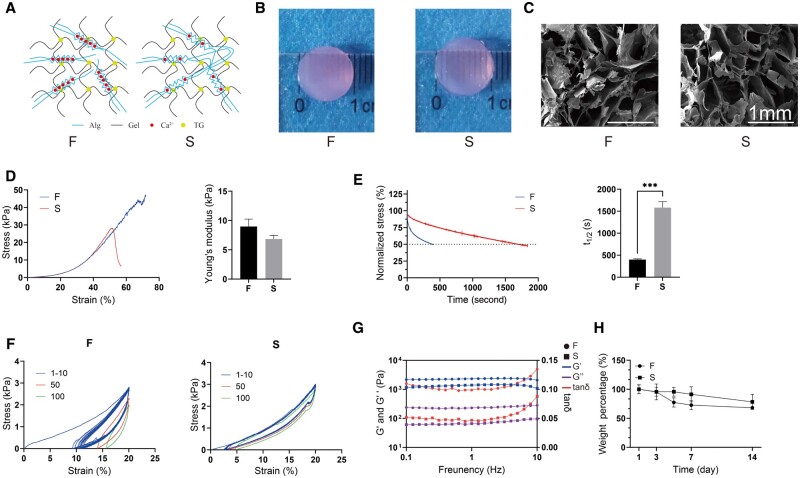
Characterization of physical properties of two hydrogels in F and S groups, respectively. (**A**) Diagram of the hydrogel structure. (**B**) Appearance diagram of the hydrogels. (**C**) Scanning electron microscopy (SEM) observations of the hydrogels. (**D**) Compressive stress–strain curves and Young's modulus of hydrogels. (**E**) Relaxation curves and relaxation times of hydrogels. (**F**) Stress–strain curves of the hydrogels under cyclic compression under maximum strain of 20%. (**G**) Rheological measurements of the hydrogels. (**H**) *In vitro* degradation curves of hydrogels. (Data are presented as the mean ± SD, *n* = 3, *P < 0.05, **P < 0.01, ***P < 0.001, ****P < 0.0001.)

The structure of two hydrogels are shown in Figure 1A. Figure 1B shows that the hydrogel was translucent after a day of equilibration in the medium, which ensured that cells could be observed growing on it under the microscope. The microstructures of the two hydrogels were very similar, and both had large pore diameters, as shown in Figure 1C. The uniaxial compressive stress–strain curves of the two hydrogels are shown in Figure 1D. The elastic moduli of the two hydrogels were 9 kPa for hydrogel F and 7 kPa for hydrogel S, respectively, the difference being not significant, indicating no difference in the initial elastic moduli between them. The stress relaxation test showed that the two hydrogels had different stress relaxation times, 399 s for hydrogel F and 1582 s for hydrogel S, thus different viscoelasticity (Figure 1E). A cyclic compression test and rheology test were performed to further explore the mechanical properties of the hydrogels. The loading and unloading curves of hydrogels F at different cycles were significantly different compared to those of the hydrogel S, and the decrease of maximum stress with increasing cycles indicated significant energy loss (Figure 1F). The loading and unloading curves of hydrogels S at different cycles were almost the same, suggesting a small energy loss. The rheological test showed that the two hydrogels have similar storage modulus (*G*′), but the loss modulus (*G*″) and loss factor (tan *δ*) of hydrogel F are higher than those of hydrogel S (Figure 1G). The results showed that the two sodium alginate–gelatine hydrogels have similar initial elastic modulus and distinct viscoelasticity. The degradation performance of the two hydrogels in the culture medium shown in Figure 1H demonstrated that hydrogel F degraded to 68% and hydrogel S degraded to 78% of the initial mass after 14 days.

### Morphology, viability and migration of cells cultured on sodium alginate–gelatine hydrogel

**Figure 2.**
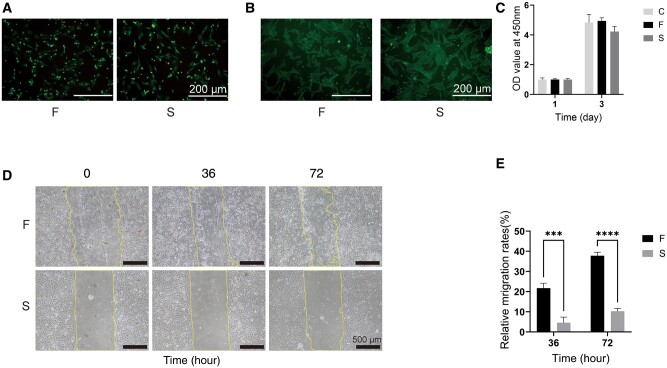
Cell morphology, proliferation and migration on the hydrogel surface in F and S groups, respectively. (**A**) Live/dead staining of cells on the hydrogel surface. (**B**) Cytoskeleton staining of BMSCS. (**C**) CCK-8 cell proliferation assay. (**D**) Images of cell scratch migration. (**E**) Quantification of cell scratch migration images. (Data are presented as the mean ± SD, *n* = 3, *P < 0.05, **P < 0.01, ***P < 0.001, ****P < 0.0001.)

The live/dead staining in Figure 2A shows the presence of few dead cells in the two groups of hydrogels, revealing a good biocompatibility, demonstrating that the materials used in the manufacturing of hydrogels were non-cytotoxic, and the crosslinker Ca sulphate and TG enzyme have little effect on cell viability [41]. The activity of cultured cells in the hydrogel extract liquor measured by CCK-8 (Figure 2C) demonstrated that the number of cells in the two groups increased after 3 days and this increase was not significantly difference compared with that of the control group. This suggested that the addition of TG enzyme in the hydrogel did not affect the growth and proliferation of cells, which was consistent with the results previously reported [42]. The cell adhesion ability of the hydrogels was evaluated by inoculating BMSCs on the hydrogels, and then, the cytoskeleton staining showed that the cells effectively adhered and diffused on the hydrogels F and S (Figure 2B). The addition of gelatine should give cell adhesion ability to the gelatine–sodium alginate hydrogels. The migration ability of cells on the two hydrogels was also detected by the scratch assay, which revealed the cells migrate faster on hydrogel F than on hydrogel S, as observed under a light microscope (Figure 2D and E).

### Fast stress relaxation hydrogels promote osteogenesis *in vitro*

**Figure 3.**
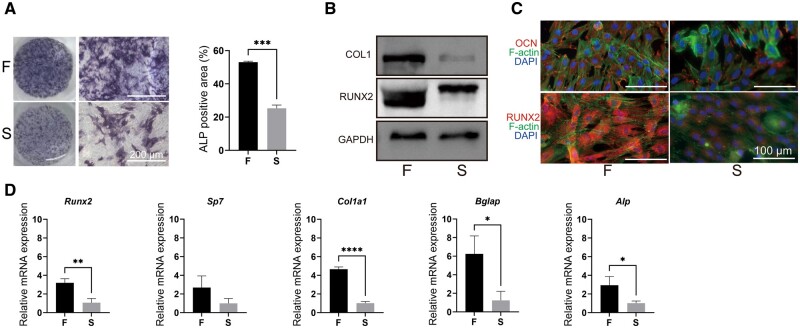
Osteogenesis of BMSCS cultured on hydrogels *in vitro*. (**A**) Images and quantification of ALP staining of BMSCs on the hydrogels. (**B**, **C**) Protein and (**D**) mRNA expression of osteogenesis-related genes in BMSCs cultured on hydrogels. (Data are presented as the mean ± SD, *n* = 3, *P < 0.05, **P < 0.01, ***P < 0.001, ****P < 0.0001.)

Alkaline phosphatase (ALP) staining and area quantification performed after 7 days of cell culture in an osteogenic medium showed that it was significantly higher in the cells cultured on hydrogel F than in the cells on hydrogel S (Figure 3A). The results of western blot in Figure 3B and immunofluorescence in Figure 3C showed that hydrogel F significantly promoted the expression of the osteogenic proteins RUNX2, COL1 and OCN. Similarly, the expressions of several osteogenic differentiation-related mRNA were also detected in BMSCs, revealing that hydrogel F significantly promoted the mRNA expressions of the osteogenesis-related genes *ALP*, *RUNX2*, *COL1a1* and *Bglap* (Figure 3D). These results strongly suggested that the viscoelasticity of hydrogel regulated the expression of osteogenic genes in BMSCs, ultimately leading to the higher osteogenic activity of BMSCs cultured on fast stress relaxation hydrogels.

### TRPV4 mediates a faster stress relaxation on osteogenic differentiation

**Figure 4.**
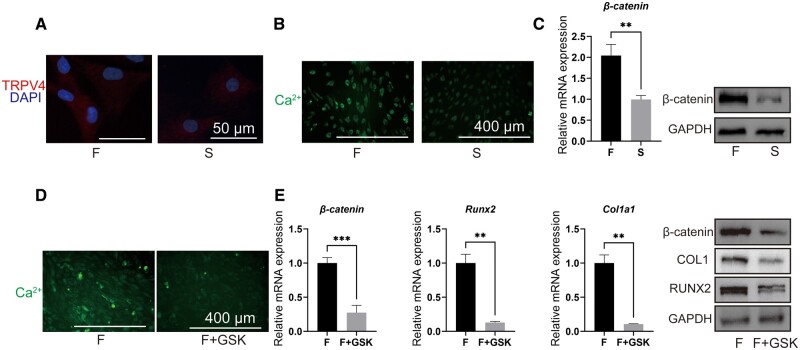
Fast stress relaxation hydrogel promotes osteogenic differentiation of BMSCs by activating TRPV4. (**A**) Immunofluorescence staining of cellular TRPV4 on hydrogels. (**B**) Fluo-4 AM staining within cells on hydrogels. (**C**) mRNA and protein expression of β-catenin in cells on hydrogels. (**D**) Fluo-4 AM staining on the fast stress relaxation hydrogel after TRPV4 inhibition. (**E**) The mRNA and protein expression of β-catenin and osteogenesis-related genes on after TRPV4 inhibition with fast stress relaxation hydrogel. (GSK stands for GSK2193874, data are presented as the mean ± SD, *n* = 3, *P < 0.05, **P < 0.01, ***P < 0.001, ****P < 0.0001.)

Immunofluorescence showed that BMSCs expressed TRPV4 on both hydrogels (Figure 4A). Fluo-4 AM staining showed that intracellular Ca^2+^ levels were higher in BMSCs cultured on hydrogel F compared with those in BMSCs cultured on hydrogel S (Figure 4B), indicating that fast stress relaxation activated TRPV4. Cells cultured on the two media were exposed to different concentrations of Ca ions because different concentrations of Ca sulphate were used in the two hydrogel manufacturing processes. The Ca ion concentration of the osteogenic induction medium immersed in the two hydrogels for 1 day was 8.2 mM in the hydrogel F, and was 2.4 mM in the hydrogel S (Supplementary Figure S2A). To rule out the effect of Ca ion concentration in the medium on intracellular Ca ion concentration on the different viscoelastic hydrogels, the hydrogel S group was adjusted to 8.2 mM using Ca chloride. The Fluo-4 AM staining results showed that the intracellular Ca ion of BMSCs on hydrogel F was still higher, proving the influence of viscoelasticity on the intracellular Ca ion concentration (Supplementary Figure S2B). Then, the role of Wnt/β-catenin pathway on the effect of TRPV4 on osteoblast differentiation of BMSCs was assessed, revealing that *β-catenin* mRNA expression was significantly increased in BMSCs cultured on hydrogel F. Western blot results were similar, since β-catenin protein expression was significantly increased in BMSCs cultured on hydrogel F (Figure 4C). Then, the pharmacologically specific inhibitor of TRPV4 (GSK2193874) was added to the medium to further determine the effect of TRPV4 activation on the osteogenic differentiation of BMSCs cultured on hydrogel F. Intracellular Ca^2+^ levels were decreased after adding GSK2193874 compared with those in BMSCs cultured on hydrogel F (Figure 4D). The expression of *β-catenin* mRNA and protein was significantly decreased after adding GSK2193874 compared with that of BMSCs cultured on hydrogel F. Moreover, the expression of the osteoblast-related mRNA *Col1a1* and *Runx2* and the osteoblast-related proteins COL1 and RUNX2 were significantly decreased (Figure 4E). These results suggested that fast stress relaxation promoted TRPV4 activation to enhance intracellular Ca^2+^, which further increased the Wnt/β-catenin signalling, leading to the upregulation of osteoblast gene expression. Thus, TRPV4 worked as a mediator of fast stress-induced osteoblast BMSC differentiation.

### Dynamic compressions on cell-laden viscoelastic hydrogels promote osteogenesis *in vitro*

**Figure 5.**
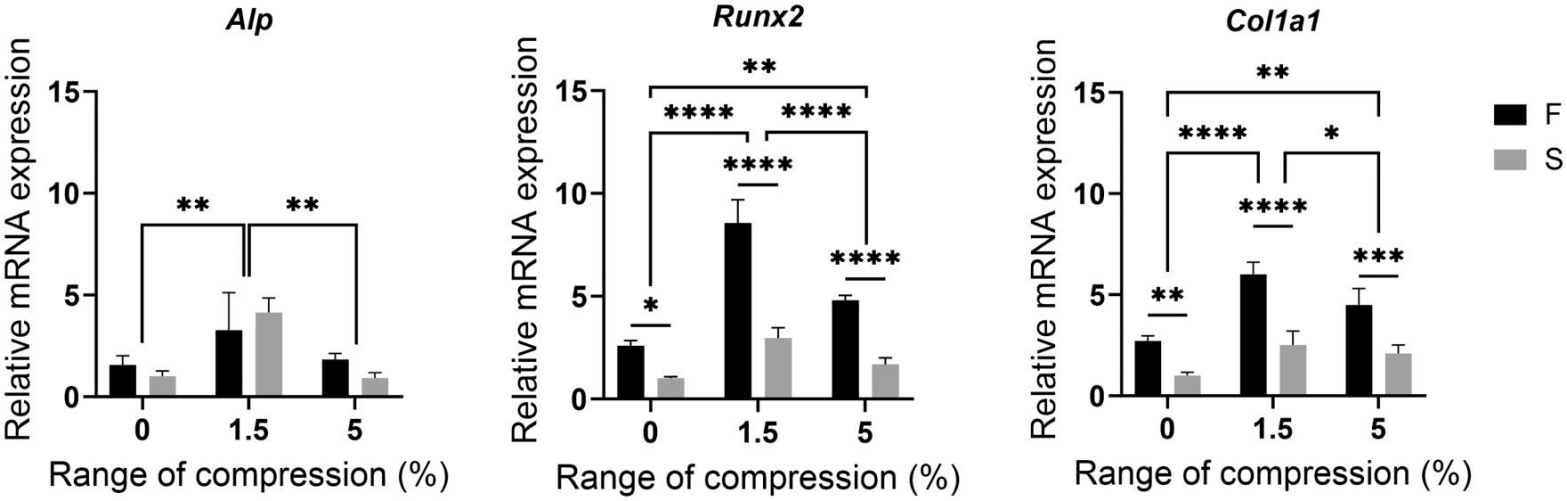
Combined effects of viscoelastic hydrogel and dynamic compressive loading on mRNA expression of osteogenesis-related genes in BMSCs. (Data are presented as the mean ± SD, *n* = 3, *P < 0.05, **P < 0.01, ***P < 0.001, ****P < 0.0001.)

The expression of osteogenesis-related mRNAs in BMSCs was enhanced in both hydrogels after 1.5% compressive strain was applied to the hydrogel (Figure 5), while the expression of osteogenesis-related mRNA in BMSCs was decreased in both hydrogels after 5% compressive strain was applied compared with that after 1.5%, although still higher than that under 0% compressive strain. In addition, the combined effect of fast stress relaxation hydrogel and compressive strain on bone formation was greater than that of either alone. Fast stress relaxation hydrogel combined with 1.5% compressive strain showed the greatest osteopromoting effect.

### Viscoelastic hydrogels promote osteogenesis *in vivo*

**Figure 6.**
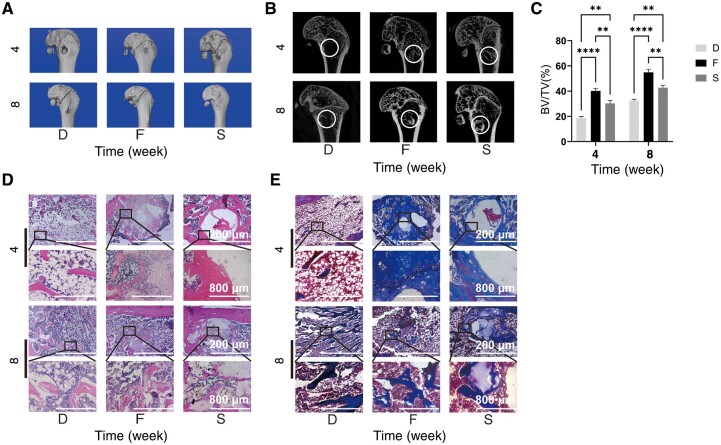
Viscoelastic hydrogel promotes bone formation *in vivo*. (**A** and **B**) Microscopic computed tomography of the femur. (**C**) Quantitative analysis of newly regenerated bone tissue. (**D**) H&E staining of the defect site at 4 and 8 weeks. (**E**) Masson staining of the defect site at weeks 4 and 8 after surgery. (Data are presented as the mean ± SD, *n* = 3, *P < 0.05, **P < 0.01, ***P < 0.001, ****P < 0.0001.)

Bone formation was assessed by implanting the two viscoelastic hydrogels into the femoral defects of rats. The results were similar at 4 and 8 weeks, with the hydrogel F showing the most bone formation and the defect group showing the least bone regeneration (Figure 6A and B). The quantification of BV/TV in the defect area also showed that hydrogel F had the highest bone volume (Figure 6C). H&E staining and Masson staining (Figure 6D and E) showed more new bones in the hydrogel F although the presence of hydrogel residues in both hydrogel F and hydrogel S groups at 4 weeks. The hydrogel F completely disappeared after 8 weeks, and the new bone trabecula structure was similar to that of the normal bone, but some hydrogel residues were present in the hydrogel S. These results demonstrated that matrix viscoelasticity effectively regulated bone formation *in vivo*.

## Discussion

Sodium alginate has long been used in tissue engineering and biological studies [43]. The viscoelasticity of alginate hydrogels can be adjusted by changing the molecular weight of the alginate polymer, changing the type of crosslinker (ionic or covalent) or linking short polyethylene glycol branches to the alginate polymer [20, 40, 44]. The elasticity modulus can also be easily adjusted by changing the amount of crosslinker, decoupled from the viscous behaviour. Therefore, alginate hydrogels are widely used in the study of viscoelastic biomaterials. However, sodium alginate itself does not have cell adhesion sites and is highly hydrophilic, making it unable to adsorb proteins well and interact directly with cells [43]. Therefore, sodium alginate is usually mixed with other substances with cell adhesion ability [45] or it is coupled with arginylglycylaspartic acid (RGD) peptides to obtain cell adhesion ability [46] and investigate the effect of viscoelasticity on cells [20, 23, 44], which was also investigate on hydrogels mixed with collagen and sodium alginate. Hydrogels prepared by mixing gelatine and sodium alginate have good cell adhesion ability. Therefore, this study aimed to find a simple and rapid method to add gelatine to sodium alginate hydrogel, without affecting the regulation of its mechanical properties, to make sodium alginate hydrogel with cell adhesion ability for the study of viscoelastic biological effects. Although there are studies exploring the growth of stem cell particles in viscoelastic gelatine sodium alginate hydrogels [47], there is no control of the elastic modulus of the hydrogel to keep it consistent. Therefore, in this study sodium alginate–gelatine hydrogel was obtained by simply mixing sodium alginate with gelatine solution, crosslinking sodium alginate with Ca sulphate, and crosslinking gelatine with TG enzyme. By changing the molecular weight of sodium alginate and the content of Ca in different types of hydrogels to control the viscoelasticity and elastic modulus, hydrogels with similar elastic modulus but different viscoelasticity were obtained. Our hydrogel raw material had good biocompatibility [20, 42], thus ensuring that it was not cytotoxic. Cells were cultured on the hydrogel using the hydrogel extract liquor and then, cell activity was detected, which was similar to our previous results showing that our hydrogel has good biocompatibility [41].

Cell migration is necessary for the regeneration of various tissues [10]. The migration of BMSCs to the bone defect site and osteogenic differentiation are also fundamental for bone regeneration [48]. Some studies on the effect of viscoelasticity on cell migration were performed. Adebowale [49] found that fast stress relaxation promotes cell migration on a soft matrix. Our hydrogels also showed similar results. The faster migration rate of BMSCs on the fast stress hydrogel suggested their potential osteopromoting ability.

Cameron [19] found that an increase in matrix loss modulus increases the osteogenic differentiation of hMSCs, probably due to an increase in the diffusion area of the cells on gels with high loss modulus. Chaudhuri [20] research shows that fast stress relaxation in the 3D culture of sodium alginate hydrogels enhances MSC cell diffusion, proliferation and osteogenic differentiation by increasing integrin adhesion, local aggregation of RGD ligands, actomyosin contraction and nuclear localization of YAP. Lee [23] demonstrates that the activation of TRPV4 ion channels and the expansion of cell volume in the 3D culture of fast stress relaxation of sodium alginate hydrogels lead to osteogenic differentiation of MSCs. Whitehead [50] demonstrated the increase of the osteogenic differentiation ability of MSC spheroids in viscoelastic sodium alginate hydrogels. Our study, in line with these results, also showed an increased osteogenic differentiation of BMSCs cultured on the surface of fast stress relaxation hydrogels. Additionally, our hydrogel material itself is also stained by Alizarin Red, this can mask the signal of calcium nodules and affect result accuracy. Therefore, we did not conduct late-stage detection of mineralized products such as calcium nodules.

TRPV4 is a Ca channel that is activated by chemical and physical stimuli, including force [51]. Force causes cytoskeletal deformation, leading to TRPV4 opening to increase Ca^2+^ influx, consequently increasing intracellular Ca levels [52]. This increase leads to the nuclear translocation of NFATc1, which induces the upregulation of Wnt/β-catenin expression and the increase in the nuclear translocation of β-catenin [53]. Intranuclear β-catenin binds to the transcription factor TCF1, thereby upregulating osteoblast gene expression. Our study found that fast stress relaxation promoted TRPV4 activation that increased Ca^2+^ influx, which was inhibited by the TRPV4 antagonist GSK2193874, which also inhibited the fast stress relaxation-induced increase in β-catenin mRNA and protein expression and the increase in osteogenic activity of BMSCs. This suggests that TRPV4 mediated fast stress-induced osteogenic differentiation of BMSCs.

Biomechanical signals are essential for bone homeostasis, healing and remodelling. Compressive strain promotes osteogenic differentiation and mineralization of cells. Rath applied 10% and 20% compressive strains to an electrospun polycaprolactone scaffold seeded with calvarial osteoblasts and that of 10% promotes better the osteogenic differentiation of cells [54]. Seo applied compression strains of 10%, 27% and 42% to the methacryl gelatine encapsulating hMSC revealing that the greater the strain, the stronger the osteogenic differentiation of the cells [55]. The compressive strains used in the above studies were large beyond the physiological deformation of the bone. Our study found that a 1.5% physiological strain produced a significant osteopromoting effect, while the effect of a larger strain was decreased. This difference may arise due to differences in the three-dimensional culture medium, where even the slow stress relaxing sodium alginate hydrogel has some viscoelasticity. The viscoelasticity may have contributed to the reduced degree of strain at which osteogenesis begins to take place. Moreover, matrix viscoelasticity significantly increased the osteogenic effect of the compressive strain.

The fast stress relaxation hydrogel possesses higher osteogenesis ability in both the rat skull defect and dorsal subcutaneous tissue [24, 50, 56]. However, no biomechanical factors are involved in bone repair and osteogenesis at these sites. Our experiments demonstrated the promotion of the osteogenic differentiation by the effect of fast stress relaxation hydrogel plus compressive strain. Due to the dynamic compressive strain during femoral defect repair, the fast stress relaxation hydrogel should achieve better results in femoral defect repair. And the fast stress relaxation hydrogel is effective in the repair of femoral osteochondral defects [57]. The present study showed that the fast stress relaxation hydrogel significantly promoted bone regeneration at the femoral defect site, which might be the joint result of fast stress relaxation inducing BMSCs migration, osteogenic differentiation and compressive strain generated by movement. These results suggested that viscoelasticity regulated bone regeneration in multiple sites. The *in vivo* results were highly consistent with the *in vitro* results, indicating the importance of matrix viscoelasticity and mechanical loading in bone regeneration and suggesting that the role of the mechanical microenvironment in tissue regeneration should be considered.

Although positive results in promoting osteogenic differentiation of BMSCs were obtained in this work by fast stress relaxation and mechanical loading, it also has some limitations. The mechanism used by the viscoelastic hydrogel to activate TRPV4 was not investigated. However, it has been shown that the volumetric expansion of MSCs in 3D fast stress hydrogel leads to the activation of TRPV4 and subsequent osteogenic differentiation [23]. Our hypothesis was that the cultured cells in 2D fast stress hydrogel might also have a larger volume which increased the membrane tension and led to the activation of TRPV4. It is insufficient to demonstrate the additive effect of fast stress relaxation and mechanical loading in promoting bone defect repair in *in vivo* experiments due to the technical limitation in designing different mechanical loads. Our future studies will explore the combined application of mechanical loading and viscoelastic biomaterials in bone defects and the involved signalling pathways *in vivo*.

## Conclusion

This study developed a sodium alginate–gelatine hydrogel with adjustable viscoelastic properties. The fast stress relaxation of the hydrogel activated the TRPV4 channel, promoting the osteogenic differentiation of BMSCs, which involved an increase in intracellular Ca levels and the activation of the Wnt/β-catenin signalling pathway. In addition, slight compressive strains also promote osteogenic differentiation, and their effect can be superimposed on rapid stress relaxation. In addition, the hydrogel promoted bone regeneration in a rat femoral defect model under natural mechanical load. These findings might not only guide the design of new biomaterials but also improve our understanding of cell–material interactions and the role of mechanical loading in tissue engineering.

## Supplementary Material

rbae136_Supplementary_Data

## References

[1] Service RF. Tissue engineers build new bone. Science 2000;289:1498–500. 10991738 10.1126/science.289.5484.1498

[2] Ding ZZ , Fan ZH, Huang XW, Lu Q, Xu WA, Kaplan DL. Silk-hydroxyapatite nanoscale scaffolds with programmable growth factor delivery for bone repair. ACS Appl Mater Interfaces 2016;8:24463–70. 27579921 10.1021/acsami.6b08180

[3] Yu W , Li R, Long J, Chen P, Hou AY, Li L, Sun X, Zheng GQ, Meng HY, Wang Y, Wang AY, Sui X, Guo QY, Tao S, Peng J, Qin L, Lu SB, Lai YX. Use of a three-dimensional printed polylactide-coglycolide/tricalcium phosphate composite scaffold incorporating magnesium powder to enhance bone defect repair in rabbits. J Orthop Translat 2019;16:62–70. 30723682 10.1016/j.jot.2018.07.007 PMC6350073

[4] Henkel J , Woodruff MA, Epari DR, Steck R, Glatt V, Dickinson IC, Choong PF, Schuetz MA, Hutmacher DW. Bone regeneration based on tissue engineering conceptions—a 21st century perspective. Bone Res 2013;1:216–48. 26273505 10.4248/BR201303002 PMC4472104

[5] Zheng J , Wang Y, Wang Y, Duan R, Liu L. Gelatin/hyaluronic acid photocrosslinked double network hydrogel with nano-hydroxyapatite composite for potential application in bone repair. Gels 2023;9:742. 37754423 10.3390/gels9090742 PMC10530748

[6] Saghati S , Avci CB, Hassani A, Nazifkerdar S, Amini H, Saghebasl S, Mahdipour M, Banimohamad-Shotorbani B, Namjoo AR, Abrbekoh FN, Rahbarghazi R, Nasrabadi HT, Khoshfetrat AB. Phenolated alginate hydrogel induced osteogenic properties of mesenchymal stem cells via Wnt signaling pathway. Int J Biol Macromol 2023;253:127209. 37804896 10.1016/j.ijbiomac.2023.127209

[7] Chen K , He W, Gao W, Wu Y, Zhang Z, Liu M, Hu Y, Li XX, Feng FQ. A dual reversible cross-linked hydrogel with enhanced mechanical property and capable of proangiogenic and osteogenic activities for bone defect repair. Macromol Biosci 2024;24:e2300325. 37805941 10.1002/mabi.202300325

[8] Liu G , Guo Q, Liu C, Bai J, Wang H, Li J, Liu D, Yu Q, Shi J, Liu C, Zhu C, Li B, Zhang H. Cytomodulin-10 modified GelMA hydrogel with kartogenin for in-situ osteochondral regeneration. Acta Biomater 2023;169:317–33. 37586447 10.1016/j.actbio.2023.08.013

[9] Jiang T , Zhao J, Yu S, Mao Z, Gao C, Zhu Y, Mao C, Zheng L. Untangling the response of bone tumor cells and bone-forming cells to matrix stiffness and adhesion ligand density by means of hydrogels. Biomaterials 2019;188:130–43. 30343256 10.1016/j.biomaterials.2018.10.015 PMC6279509

[10] Vining KH , Mooney DJ. Mechanical forces direct stem cell behavior in development and regeneration. Nat Rev Mol Cell Biol 2017;18:728–42. 29115301 10.1038/nrm.2017.108 PMC5803560

[11] Wang N , Tytell JD, Ingber DE. Mechanotransduction at a distance: mechanically coupling the extracellular matrix with the nucleus. Nat Rev Mol Cell Biol 2009;10:75–82. 19197334 10.1038/nrm2594

[12] Discher DE , Janmey P, Wang YL. Tissue cells feel and respond to the stiffness of their substrate. Science 2005;310:1139–43. 16293750 10.1126/science.1116995

[13] Pelham RJ , Wang Y. Cell locomotion and focal adhesions are regulated by substrate flexibility. Proc Natl Acad Sci U S A 1997;94:13661–5. 9391082 10.1073/pnas.94.25.13661 PMC28362

[14] Klein EA , Yin L, Kothapalli D, Castagnino P, Byfield FJ, Xu T, Levental I, Hawthorne E, Janmey PA, Assoian RK. Cell-cycle control by physiological matrix elasticity and in vivo tissue stiffening. Curr Biol 2009;19:1511–8. 19765988 10.1016/j.cub.2009.07.069 PMC2755619

[15] Engler AJ , Sen S, Sweeney HL, Discher DE. Matrix elasticity directs stem cell lineage specification. Cell 2006;126:677–89. 16923388 10.1016/j.cell.2006.06.044

[16] Engler A , Bacakova L, Newman C, Hategan A, Griffin M, Discher D. Substrate compliance versus ligand density in cell on gel responses. Biophys J 2004;86:617–28. 14695306 10.1016/S0006-3495(04)74140-5 PMC1303831

[17] Dembo M , Wang YL. Stresses at the cell-to-substrate interface during locomotion of fibroblasts. Biophys J 1999;76:2307–16. 10096925 10.1016/S0006-3495(99)77386-8 PMC1300203

[18] Charrier EE , Pogoda K, Wells RG, Janmey PA. Control of cell morphology and differentiation by substrates with independently tunable elasticity and viscous dissipation. Nat Commun 2018;9:449. 29386514 10.1038/s41467-018-02906-9 PMC5792430

[19] Cameron AR , Frith JE, Cooper-White JJ. The influence of substrate creep on mesenchymal stem cell behavior and phenotype. Biomaterials 2011;32:5979–93. 21621838 10.1016/j.biomaterials.2011.04.003

[20] Chaudhuri O , Gu L, Klumpers D, Darnell M, Bencherif SA, Weaver JC, Huebsch N, Lee HP, Lippens E, Duda GN, Mooney DJ. Hydrogels with tunable stress relaxation regulate stem cell fate and activity. Nat Mater 2016;15:326–34. 26618884 10.1038/nmat4489 PMC4767627

[21] Chaudhuri O , Gu L, Darnell M, Klumpers D, Bencherif SA, Weaver JC, Huebsch N, Mooney DJ. Substrate stress relaxation regulates cell spreading. Nat Commun 2015;6:6364. 10.1038/ncomms7365 PMC4518451 25695512

[22] Gong Z , Szczesny SE, Caliari SR, Charrier EE, Chaudhuri O, Cao X, Lin Y, Mauck RL, Janmey PA, Burdick JA, Shenoy VB. Matching material and cellular timescales maximize cell spreading on viscoelastic substrates. Proc Natl Acad Sci U S A 2018;115:E2686–95. 29507238 10.1073/pnas.1716620115 PMC5866566

[23] Lee HP , Stowers R, Chaudhuri O. Volume expansion and TRPV4 activation regulate stem cell fate in three-dimensional microenvironments. Nat Commun 2019;10:529. 30705265 10.1038/s41467-019-08465-x PMC6355972

[24] Darnell M , Young S, Gu L, Shah N, Lippens E, Weaver J, Duda G, Mooney D. Substrate stress-relaxation regulates scaffold remodeling and bone formation in vivo. Adv Healthc Mater 2017;6:10. 10.1002/adhm.201601185 PMC5440842 27995768

[25] Huang DY , Li YH, Ma ZH, Lin H, Zhu XD, Xiao Y, Zhang XD. Collagen hydrogel viscoelasticity regulates MSC chondrogenesis in a ROCK-dependent manner. Sci Adv 2023;9:eade9497. 36763657 10.1126/sciadv.ade9497 PMC9916999

[26] Nikukar H , Reid S, Tsimbouri PM, Riehle MO, Curtis ASG, Dalby MJ. Osteogenesis of mesenchymal stem cells by nanoscale mechanotransduction. ACS Nano 2013;7:2758–67. 23442213 10.1021/nn400202j

[27] Uzer G , Pongkitwitoon S, Ete Chan M, Judex S. Vibration-induced osteogenic commitment of mesenchymal stem cells is enhanced by cytoskeletal remodeling but not fluid shear. J Biomech 2013;46:2296–302. 23870506 10.1016/j.jbiomech.2013.06.008 PMC3777744

[28] Evans FG. Factors affecting the mechanical properties of bone. Bull N Y Acad Med 1973;49:751–64. 4517771 PMC1807062

[29] Tomlinson RE , Li Z, Li Z, Minichiello L, Riddle RC, Venkatesan A, Clemens TL. NGF-TrkA signaling in sensory nerves is required for skeletal adaptation to mechanical loads in mice. Proc Natl Acad Sci U S A 2017;114:E3632–41. 28416686 10.1073/pnas.1701054114 PMC5422802

[30] McGarry JG , Klein-Nulend J, Mullender MG, Prendergast PJ. A comparison of strain and fluid shear stress in stimulating bone cell responses—a computational and experimental study. FASEB J 2005;19:482–4. 15625080 10.1096/fj.04-2210fje

[31] Neidlinger-Wilke C , Wilke HJ, Claes L. Cyclic stretching of human osteoblasts affects proliferation and metabolism: a new experimental method and its application. J Orthop Res 1994;12:70–8. 8113944 10.1002/jor.1100120109

[32] Tang L , Lin Z, Li YM. Effects of different magnitudes of mechanical strain on osteoblasts in vitro. Biochem Biophys Res Commun 2006;344:122–8. 16603128 10.1016/j.bbrc.2006.03.123

[33] Strotmann R , Harteneck C, Nunnenmacher K, Schultz G, Plant TD. OTRPC4, a nonselective cation channel that confers sensitivity to extracellular osmolarity. Nat Cell Biol 2000;2:695–702. 11025659 10.1038/35036318

[34] O'Conor CJ , Griffin TM, Liedtke W, Guilak F. Increased susceptibility of Trpv4-deficient mice to obesity and obesity-induced osteoarthritis with very high-fat diet. Ann Rheum Dis 2013;72:300–4. 23178209 10.1136/annrheumdis-2012-202272 PMC3549299

[35] Kang SS , Shin SH, Auh CK, Chun J. Human skeletal dysplasia caused by a constitutive activated transient receptor potential vanilloid 4 (TRPV4) cation channel mutation. Exp Mol Med 2012;44:707–22. 23143559 10.3858/emm.2012.44.12.080 PMC3538978

[36] Matthews BD , Thodeti CK, Tytell JD, Mammoto A, Overby DR, Ingber DE. Ultra-rapid activation of TRPV4 ion channels by mechanical forces applied to cell surface beta1 integrins. Integr Biol (Camb) 2010;2:435–42. 20725677 10.1039/c0ib00034e PMC3147167

[37] Lee KL , Guevarra MD, Nguyen AM, Chua MC, Wang Y, Jacobs CR. The primary cilium functions as a mechanical and calcium signaling nexus. Cilia 2015;4:7. 26029358 10.1186/s13630-015-0016-y PMC4448211

[38] Corrigan MA , Johnson GP, Stavenschi E, Riffault M, Labour MN, Hoey DA. TRPV4-mediates oscillatory fluid shear mechanotransduction in mesenchymal stem cells in part via the primary cilium. Sci Rep 2018;8:3824. 29491434 10.1038/s41598-018-22174-3 PMC5830574

[39] Hu K , Sun H, Gui B, Sui C. TRPV4 functions in flow shear stress induced early osteogenic differentiation of human bone marrow mesenchymal stem cells. Biomed Pharmacother 2017;91:841–8. 28501773 10.1016/j.biopha.2017.04.094

[40] Charbonier F , Indana D, Chaudhuri O. Tuning viscoelasticity in alginate hydrogels for 3D cell culture studies. Curr Protoc 2021;1:e124. 10.1002/cpz1.124 PMC8171168 34000104

[41] Wen C , Lu LL, Li XS. Mechanically robust gelatin–alginate IPN hydrogels by a combination of enzymatic and ionic crosslinking approaches. Macro Mater Eng 2014;299:504–13.

[42] Xu XW , Li XY, Qiu S, Zhou Y, Li L, Chen X, Zheng K, Xu Y. Concentration selection of biofriendly enzyme-modified gelatin hydrogels for periodontal bone regeneration. ACS Biomater Sci Eng 2023;9:4341–55. 37294274 10.1021/acsbiomaterials.3c00166

[43] Rowley JA , Madlambayan G, Mooney DJ. Alginate hydrogels as synthetic extracellular matrix materials. Biomaterials 1999;20:45–53. 9916770 10.1016/s0142-9612(98)00107-0

[44] Nam S , Stowers R, Lou J, Xia Y, Chaudhuri O. Varying PEG density to control stress relaxation in alginate-PEG hydrogels for 3D cell culture studies. Biomaterials 2019;200:15–24. 30743050 10.1016/j.biomaterials.2019.02.004 PMC6463514

[45] Ansari S , Sarrion P, Hasani-Sadrabadi MM, Aghaloo T, Wu BM, Moshaverinia A. Regulation of the fate of dental-derived mesenchymal stem cells using engineered alginate-GelMA hydrogels. J Biomed Mater Res A 2017;105:2957–67. 28639378 10.1002/jbm.a.36148 PMC5623163

[46] Amin ML , Deng K, Tran HA, Singh R, Rnjak-Kovacina J, Thorn P. Glucose-dependent insulin secretion from beta cell spheroids is enhanced by embedding into softer alginate hydrogels functionalised with RGD peptide. Bioengineering (Basel) 2022;9:722. 36550929 10.3390/bioengineering9120722 PMC9774350

[47] Lemarie L , Dargar T, Grosjean I, Gache V, Courtial EJ, Sohier J. Human induced pluripotent spheroids' growth is driven by viscoelastic properties and macrostructure of 3D hydrogel environment. Bioengineering (Basel) 2023;10:1418. 38136009 10.3390/bioengineering10121418 PMC10740696

[48] Du B , Liu W, Deng Y, Li S, Liu X, Gao Y, Zhou L. Angiogenesis and bone regeneration of porous nano-hydroxyapatite/coralline blocks coated with rhVEGF165 in critical-size alveolar bone defects in vivo. Int J Nanomedicine 2015;10:2555–65. 25848271 10.2147/IJN.S78331 PMC4386782

[49] Adebowale K , Gong Z, Hou JC, Wisdom KM, Garbett D, Lee HP, Nam S, Meyer T, Odde DJ, Shenoy VB, Chaudhuri O. Enhanced substrate stress relaxation promotes filopodia-mediated cell migration. Nat Mater 2021;20:1290–9. 33875851 10.1038/s41563-021-00981-w PMC8390443

[50] Whitehead J , Griffin KH, Gionet-Gonzales M, Vorwald CE, Cinque SE, Leach JK. Hydrogel mechanics are a key driver of bone formation by mesenchymal stromal cell spheroids. Biomaterials 2021;269:120607. 33385687 10.1016/j.biomaterials.2020.120607 PMC7870573

[51] Zeng ML , Kong S, Chen TX, Peng BW. Transient receptor potential vanilloid 4: a double-edged sword in the central nervous system. Mol Neurobiol 2023;60:1232–49. 36434370 10.1007/s12035-022-03141-6

[52] Jiang D , Guo R, Dai R, Knoedler S, Tao J, Machens HG, Rinkevich Y. The multifaceted functions of TRPV4 and calcium oscillations in tissue repair. Int J Mol Sci 2024;25:1179. 38256251 10.3390/ijms25021179 PMC10816018

[53] Saidak Z , Haÿ E, Marty C, Barbara A, Marie PJ. Strontium ranelate rebalances bone marrow adipogenesis and osteoblastogenesis in senescent osteopenic mice through NFATc/Maf and Wnt signaling. Aging Cell 2012;11:467–74. 22321691 10.1111/j.1474-9726.2012.00804.x

[54] Rath B , Nam J, Knobloch TJ, Lannutti JJ, Agarwal S. Compressive forces induce osteogenic gene expression in calvarial osteoblasts. J Biomech 2008;41:1095–103. 18191137 10.1016/j.jbiomech.2007.11.024 PMC2291547

[55] Seo J , Shin JY, Leijten J, Jeon O, Bal Ozturk A, Rouwkema J, Li Y, Shin SR, Hajiali H, Alsberg E, Khademhosseini A. Interconnectable dynamic compression bioreactors for combinatorial screening of cell mechanobiology in three dimensions. ACS Appl Mater Interfaces 2018;10:13293–303. 29542324 10.1021/acsami.7b17991 PMC6939619

[56] Kim SH , Thambi T, Giang Phan VH, Lee DS. Modularly engineered alginate bioconjugate hydrogel as biocompatible injectable scaffold for in situ biomineralization. Carbohydr Polym 2020;233:115832. 32059885 10.1016/j.carbpol.2020.115832

[57] Liu C , Yu Q, Yuan Z, Guo Q, Liao X, Han F, Feng T, Liu G, Zhao R, Zhu Z, Mao H, Zhu C, Li B. Engineering the viscoelasticity of gelatin methacryloyl (GelMA) hydrogels via small "dynamic bridges" to regulate BMSC behaviors for osteochondral regeneration. Bioact Mater 2023;25:445–59. 37056254 10.1016/j.bioactmat.2022.07.031 PMC10087107

