# Supplementary material for: Viscoelastic hydrogel combined with dynamic compression promotes osteogenic differentiation of bone marrow mesenchymal stem cells and bone repair in rats
Source: Regen Biomater. 2024 Nov 23;12:rbae136. doi: 10.1093/rb/rbae136 (PMC11751691; doi:10.1093/rb/rbae136)
Supplement: rbae136_Supplementary_Data [file rbae136_supplementary_data.docx]

**Supplementary**

Table1. Hydrogel formulation

| Sample | MW of alginate | Total gel volume (μL) | Syringe 1 | Syringe 2 | | | Final concentrations | | | | Mechanical properties | |
| --- | --- | --- | --- | --- | --- | --- | --- | --- | --- | --- | --- | --- |
|  |  |  | Volume of 3% (w/v) alginate/3% (w/v) gelatin | Volume of 600 mM calcium stock (μL) | Volume of PBS (μL) | Volume of 0.5u/μL tg enzyme | Crosslinker concentration (mM) | Gelatin concentration (w/v, %) | Alginate concentration (w/v, %) | TG enzyme concentration ( u/μL ) | Initial elastic modulus  (kPa) | Stress relaxation halftime  (s) |
| Fast stress relaxation | low | 3600 | 2400 | 480 | 684 | 36 | 80 | 2 | 2 | 5 | 8.97 ± 1.26 | 399 ± 36.34 |
| Slow stress relaxation | high | 3600 | 2400 | 120 | 1044 | 36 | 20 | 2 | 2 | 5 | 6.83± 0.64 | 1581.5± 272.6 |

Table2. Primer sequence

| Gene name | Forward | Reverse |
| --- | --- | --- |
| *Gapdh* | AGTGCCAGCCTCGTCTCATA | GATGGTGATGGGTTTCCCGT |
| *Col1a1* | GTACATCAGCCCAAACCCCA | TCGCTTCCATACTCGAACTGG |
| *Bglap* | GCAGACCTAGCAGACACCAT | TTGGACATGAAGGCTTTGTCA |
| *Runx2* | CCGAGCTACGAAATGCCTCT | TGAAACTCTTGCCTCGTCCG |
| *Sp7* | GGCTGAGGAAGAAGCCCATT | AAGTGGGCTTTCAGATGCGA |
| *β-catenin* | GAAAATGCTTGGGTCGCCAG | CGCACTGCCATTTTAGCTCC |
| *Alp* | TCCTTAGGGCCACCGCT | TAATTGACGTTCCGATCCTGC |


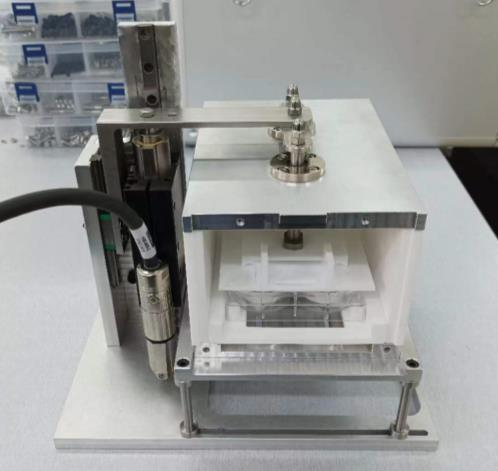


Figure. 1. Compression device for hydrogel laden with BMSCs.


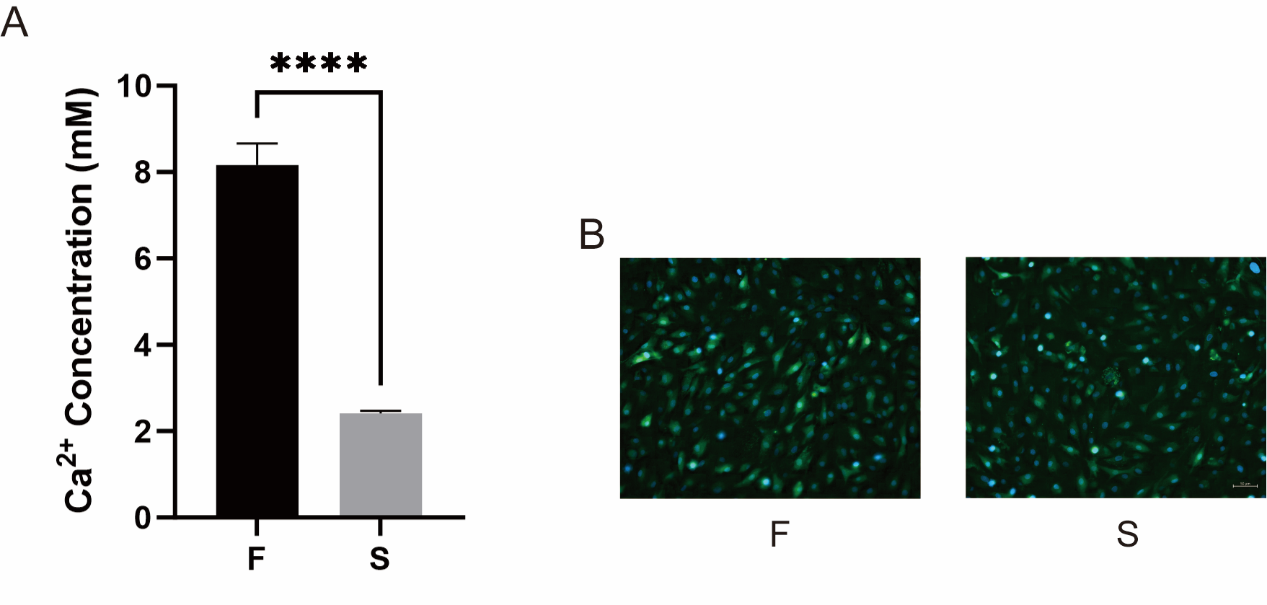


Figure. 2. (A) Calcium ion concentration in the culture medium of different groups. (B) The Fluo-4 AM staining at 8.2mM calcium in the medium. (Data are presented as the mean ± SD. ****P < 0.0001)
